# Introducing and Evaluating the Effectiveness of Online Cognitive Behavior Therapy for Gambling Disorder in Routine Addiction Care: Comparative Cohort Study

**DOI:** 10.2196/54754

**Published:** 2024-09-18

**Authors:** Olof Molander, Anne H Berman, Miriam Jakobson, Mikael Gajecki, Hanna Hällström, Jonas Ramnerö, Johan Bjureberg, Per Carlbring, Philip Lindner

**Affiliations:** 1 Centre for Psychiatry Research, Department of Clinical Neuroscience Karolinska Institutet Stockholm Health Care Services, Region Stockholm Stockholm Sweden; 2 Department of Psychology Uppsala University Uppsala Sweden; 3 Stockholm Centre for Dependency Disorders Stockholm Health Care Services, Region Stockholm Stockholm Sweden; 4 Department of Psychology Stockholm University Stockholm Sweden

**Keywords:** gambling disorder, internet-delivered cognitive behavioral therapy, routine addiction care, registry study, gambling, addiction, health care setting, iCBT, Sweden, feasibility, clinic, hospital, psychological treatment, digital intervention, addictive disorder, eHealth, digital care, survival analysis, comorbidity

## Abstract

**Background:**

Several treatment-related challenges exist for gambling disorder, in particular at-scale dissemination in health care settings.

**Objective:**

This study describes the introduction of a newly developed internet-delivered cognitive behavioral therapy (iCBT) program for gambling disorder (GD), provided with therapist support in routine addiction care, in a nationally recruited sample in Sweden. The study details the introduction of the iCBT program, evaluates its effectiveness and acceptability, and compares registry outcomes among iCBT patients with other patients with GD at the clinic who received face-to-face psychological treatment as usual.

**Methods:**

The study site was the Stockholm Addiction eClinic, which offers digital interventions for addictive disorders in routine care. The iCBT program was introduced nationally for treatment-seeking patients through the Swedish eHealth platform. After approximately 2 years of routine treatment provision, we conducted a registry study, including ordinary patients in routine digital care (n=218), and a reference sample receiving face-to-face psychological treatment for GD (n=216).

**Results:**

A statistically significant reduction in the Gambling Symptom Assessment Scale scores during the treatment was observed (B=–1.33, SE=0.17, *P*<.001), corresponding to a large within-group Cohen *d* effect size of *d*=1.39. The iCBT program was rated high for satisfaction. A registry-based survival analysis, controlling for psychiatric comorbidity, showed that patients receiving iCBT exhibited posttreatment outcomes (re-engagement in outpatient addiction care, receiving new psychiatric prescriptions, enrollment in psychiatric inpatient care, and care events indicative of contact with social services) similar to comparable patients who underwent face-to-face treatment-as-usual.

**Conclusions:**

A lack of randomized allocation notwithstanding, the iCBT program for GD evaluated in this study was well-received by patients in routine addiction care, was associated with the expected symptom decrease during treatment, and appears to result in posttreatment registry outcomes similar to face-to-face treatment. Future studies on treatment mechanisms and moderators are warranted.

**International Registered Report Identifier (IRRID):**

RR2-10.1186/s40814-020-00647-5

## Introduction

Previous-year population prevalence of problem gambling varies between 0.3% and 5.3% across countries [1] and in Sweden has been estimated to 2.1% [2]. Negative consequences related to problem gambling may occur in important life domains such as finances, relationships, or health, including a high rate of suicide ideation and attempts [3-5]. Problem gambling is a broad public health-based term, generally associated with poor mental and physical well-being. From a health care perspective, problem gambling behavior is subsumed under the gambling disorder (GD) diagnosis, formerly defined as an impulsive disorder, but included among addictive disorders at the introduction of the *Diagnostic and Statistical Manual of Mental Disorders*, fifth edition (*DSM-5*) [6]. The *DSM-5* specifies 9 diagnostic criteria for GD, observed within the past year. If at least 4 criteria are endorsed, the disorder is graded as mild (4-5 criteria), moderate (6-7), or severe (8-9) [6].

In Sweden, national prevalence rates, in combination with early studies indicating a higher problem gambling prevalence in social services and primary care samples [7,8], have led to political action. Since 2019, Sweden has introduced a semiopen gambling market, regulated by the Swedish Gambling Authority. Currently, there are 45 providers with commercial betting licenses and 56 with nonexclusive internet-based gambling licenses. These forms of gambling are most common among Swedish individuals with GD [9]. Licensed providers are required by law to provide responsible gambling tools. The primary responsible gambling tool is the Spelpaus.se voluntary self-exclusion registry, which allows gamblers to exclude themselves from all licensed land-based and internet-based gambling for various durations [10]. A recent survey from the Swedish Gambling Authority [11] indicates that 93% of daily gamblers are aware of Spelpaus.se. However, up to 30% of daily gamblers intentionally gamble with nonlicensed providers, which do not have the same duty of care obligations, including mandatory checks with the Spelpaus.se registry [11]. Furthermore, research shows that almost 40% of gamblers with an active Spelpaus.se self-exclusion continue to gamble with unlicensed providers [9].

Significant new legislation was also enacted in Sweden in 2018, which stipulated obligatory provision of GD treatment within health care and social services [12]. In recent years, evidence has accumulated for the effectiveness of psychological treatments, in particular cognitive behavioral therapy (CBT) [13]. CBT has been found effective in reducing gambling behavior and related problems in face-to-face individual and group therapies [14,15]. A recent meta-analysis also indicates lower, but promising effects for digitally delivered GD treatment [13]. In internet-delivered cognitive behavioral therapy (iCBT), treatment content is made accessible to the patient through a digital platform. The iCBT has been shown to be effective for a wide range of psychiatric as well as somatic disorders, where effects have shown equivalence to face-to-face delivered CBT [16]. Advantages specific to the internet format include cost-effectiveness, wide accessibility, scalability, and reducing perceived barriers to seeking treatment due to, for example, shame and stigma [17,18].

From a treatment dissemination and provision perspective, challenges remain. While introduction into regular health care is an obvious final goal for internet-based psychological treatment forms in general and is emphasized in treatment development models [19] and national eHealth strategies, few treatments that have been scientifically developed and evaluated are later introduced in routine health care settings and disseminated among regular patients (a study by Titov et al [20] shows an analysis of implementation of iCBT for anxiety and depression in routine care). Specific challenges exist regarding internet-based treatment for GD. First, most clinical trials evaluating iCBT for GD have been conducted in research settings, with participants recruited from the general population [17]. Second, although CBT and iCBT can be considered evidence-based treatments, the GD research field has not yet reached the same level of maturity as for other disorders [21]. Current treatments for GD often consist of a broad mix of general CBT interventions found effective for other psychiatric and addictive disorders (a study by Gooding and Tarrier [22] shows a review of GD treatment components) but lack a solid theoretical base in relation to gambling behavior [21]. It has not been understood how GD-related behavior is maintained in terms of clinical presentation, and researchers have recommended aligning treatment approaches with basic behavioral research [23,24]. However, current treatment manuals typically do not take contextual factors into consideration, including clinically pertinent factors such as exposure to gambling advertisements, low barriers to engage internet gambling, use of self-exclusion registries, and similar strategies. Third, GD is associated with high levels of psychiatric comorbidity [25]. In a meta-analysis, Dowling et al [26], found that 75% of treatment-seeking individuals with GD fulfilled an additional current *Diagnostic and Statistical Manual of Mental Disorders*, 4th edition (*DSM-IV*) Axis 1 diagnosis, such as mood disorders, alcohol use disorders, anxiety disorders, and substance use disorders. Addressing this high prevalence of psychiatric comorbidity is a pressing treatment-related GD challenge.

This study describes the introduction and subsequent effectiveness evaluation of a newly developed iCBT GD program provided with therapist support through asynchronous secure messages, within routine addiction care. The aims of the study were to (1) estimate the effectiveness and acceptability of the iCBT in a registry sample and (2) compare registry outcomes among iCBT patients with other patients with GD at the clinic who received face-to-face psychological treatment-as-usual (TAU).

## Methods

### Setting, Study Design, and Ethics

This study was conducted at the Stockholm Addiction eClinic, a specialized unit within the Stockholm Centre for Dependency Disorders offering internet-delivered psychological treatments for addictive disorders to patients with alcohol [27] or cannabis use disorders [28]. In 2019, the treatment repertoire was expanded to include GD. When the GD treatment program was introduced as part of routine care, feasibility data were initially collected in a small sample. After clinical quality assurance revealed satisfactory outcomes, a decision was made to continue to offer iCBT within the context of routine care. After approximately 2 years of routine treatment provision, a registry study was conducted on routinely collected data. A parallel cohort design was used, comparing patients in the iCBT GD treatment to patients who had received TAU at regular face-to-face outpatient clinics at the Stockholm Centre for Dependency Disorders.

At the Stockholm Centre for Dependency Disorders, patients first seek treatment for GD, either through clinical or self-referral, are clinically assessed, and choose either face-to-face treatment (for a description on the clinical treatment seeking process, see [29]) or iCBT. Both iCBT and face-to-face treatments are intended to be standalone treatments.

### Interventions

A novel iCBT program, grounded in learning theory, experimental behavioral research, and findings from interviews with patient representatives [21,24], was developed as part of a larger project [30] which aimed to conceptualize a clinical framework for the development and maintenance of GD. Briefly, the iCBT program emphasized 4 psychological processes relevant to GD. First, “access to money” is a key trigger for gambling behavior. Second, individuals with GD are likely to intensify their gambling behavior due to an exceedingly increased "reward expectancy". Third, “chasing behaviors” can be performed to chase wins and losses, and autopilot-chasing renders gambling behavior insensitive to actual aversive consequences. Fourth, “the gambling zone,” a psychological space where everything outside the gambling experience becomes irrelevant to gamblers as they become completely absorbed by the game [31], reinforces gambling behavior in itself, as well as an inability to terminate gambling once engaged.

The iCBT program consisted of 1 assessment module and 9 subsequent treatment modules, developed to encompass a simple, delimited set of core treatment components presumed to be of greatest importance for achieving control over problematic gambling behavior. Briefly, the program included psychoeducation on the 4 above-mentioned psychological processes. Patients were first instructed to register individual gambling-related situations. Thereafter, a treatment rationale was presented, emphasizing increased control over gambling behavior by completion of various behavioral exercises in gambling-related situations, in which patients were encouraged to act differently or in ways opposite than before to widen their behavioral repertoire and challenge presumptions. Patients then rated various behavioral exercises in a difficulty-rating task. After this, they performed repeated behavioral exercises with increasing difficulty, under the supervision of their assigned therapist (a licensed clinical psychologist) through weekly asynchronous secure platform messages. In these continuous messages, the iCBT therapists monitored treatment engagement and progress, provided personalized feedback on tasks, and were available to answer questions. If need be, the iCBT therapists contacted the patients, who in turn could also contact their iCBT therapist by telephone. In the beginning of the iCBT program, a standard information text instructed the patients to complete the treatment in 16-18 weeks, but time extensions were allowed if deemed clinically appropriate by the iCBT therapists.

TAU consisted of systematic psychological treatments delivered face-to-face at any of the outpatient clinics at the Stockholm Center for Dependency Disorders (nonsystematic interventions such as support conversations were excluded). Current GD treatments often lack a solid theoretical base in relation to gambling behavior [21], as suggested by the 4 central GD processes in the iCBT program. The face-to-face treatments delivered in routine addiction care therefore included a range of disparate methodological approaches and interventions, such as CBT, cognitive therapy, motivational interviewing, relapse prevention, and psychodynamic therapy, with the following national treatment provision codes: DU008, DU009, DU010, DU011, DU013, DU020, DU043, DU113, DU118, DU119, and DU120.

### Recruitment Criteria

Standard clinical eligibility criteria were used to maximize external validity. All treatment-seeking gamblers with problem gambling or GD during the past year, who were deemed clinically suitable, were offered iCBT; no prospective patient was excluded exclusively on the grounds of psychiatric comorbidities. Since patients were recruited from a clinical setting, it was not possible to accurately log how many were exposed to the offer of iCBT but turned it down.

### Treatment Introduction and Feasibility Study

The iCBT program was made available using the Swedish national eHealth platform (Stöd och behandling, Treatment and Support [TAS] platform). The TAS platform is integrated into the national health care guide 1177.se, allowing prospective patients to conveniently access information, patient services, and internet-based treatments within the same platform. Access required a bank-issued digital ID, after which notifications by SMS text message and email could be enabled by each individual user. During this first phase of the study, a feasibility study was conducted among 23 patients (“Feasibility study” in Multimedia Appendix 1 [32,33]). When the feasibility study was terminated in July 2020, clinical quality assurance work and preliminary effects indicated promising results, and the provision of the iCBT continued within the framework of regular specialist addiction care.

### Registry Study

After approximately 2 years of routine treatment provision, data from iCBT patients starting between July 2020 and August 2022 were compiled using a stepwise procedure to create a registry cohort. First, data were exported from the TAS platform that included all iCBT patients finalized at least 3 months before data export, in order to provide a minimal follow-up period for the registry outcomes. This resulted in 218 participants remaining, after removal of duplicates and 1 mislabeled test patient. Since duplicates could only result from a given patient starting and ending the program multiple times, we opted to keep only one enrollment per patient in order to approximate the intention-to-treat principle; the first treatment enrollment was selected if the TAS log file contained any activity (3 cases out of 4). Next, a health care informatician identified up to 3 reference patients for each included iCBT patient (randomly selected in case of >3 or more available) based on age (±5 years) and gender. Only iCBT patients residing in Stockholm County were matched with reference patients, to avoid introducing possible bias clustered on region of residence, that is, all patients had the same theoretical opportunity to engage in new outpatient addiction care in Region Stockholm during the registry outcome period. Raw registry data were compiled for both arms as per the description below and were then shared with the researchers. While the raw reference sample included 488 patients, 193 were subsequently excluded due to a parallel, documented care commitment at the Addiction eClinic at any time during the examined time frame, leaving 295 reference patients. Next, only reference patients who had a care event marked as constituting a systematic psychological treatment (provision codes DU008, DU009, DU010, DU011, DU013, DU020, DU043, DU113, DU118, DU119, and DU120) were retained, leaving 225 reference patients. An additional 6 reference patients were then excluded due to having care events only before 2019. This exclusion criterion was applied to better correspond with the same point in time during which the iCBT patients were undergoing treatment; this included new gambling market legislation coming into effect in 2019 in Sweden, including introduction of a national self-exclusion registry used by licensed gambling providers. Finally, 3 patients in the reference group were excluded for being <18 years, the age required for licensed gambling in Sweden, and the age limit applied at the iCBT clinic. Demographic characteristics are given in Table 1.

**Table 1:** Participant characteristics.

		iCBT^a^		TAU^b^	
		Total (n=218)	Stockholm-residing (n=123)	Total (n=216)	Matched (n=123)
Age (years), mean (SD)		36.3 (10.5)	36.1 (10.1)	37.8 (10.6)	37.3 (10.7)
**Gender, n (%)**					
	Men	167 (77)	96 (78)	175 (81)	102 (83)
	Women	51 (23)	27 (22)	41 (19)	21 (17)
**Diagnostic codes^c^, n (%)**					
	Gambling disorder (F630)	213 (98)	119 (97)	203 (94)	117 (95)
	Other *ICD*^d^ diagnoses	29 (13)	22 (18)	84 (39)	21 (17)
	Mental and behavioral disorders due to use of psychoactive substances	19 (9)	15 (12)	51 (24)	17 (14)
	Mood/affective disorders Neurotic, stress-related and somatoform disorders	5 (2)	4 (3)	29 (13)	3 (2)
		5 (2)	4 (3)	29 (13)	4 (3)
	Disorders of personality and behavior in adult persons	3 (1)	2 ()	6 (3)	1 (1)
**Previous treatments^e^, n (%)**					
	Psychological treatment in outpatient addiction care^f^	12 (6)	11 (9)	20 (9)	7 (6)
	Prescriptions of psychiatric medication in addiction care	11 (5)	11 (9)	44 (20)	10 (8)
	Psychiatric inpatient enrollment Involvement of social services	2 (1)	2 (2)	13 (6)	2 (2)
		1 (0)	1 (1)	14 (6)	1 (1)

^a^iCBT: internet-based cognitive behavioral treatment at the Stockholm Addiction eClinic. Non-Stockholm patients were excluded in the Stockholm sample.

^b^TAU: treatment-as-usual, that is, psychological treatment delivered face-to-face at any of the outpatient clinics at the Stockholm Center for Dependency Disorders. Patients at the eAddiction Clinic and non-Stockholm patients were excluded.

^c^Diagnoses linked to care-events; 2 years before treatment start to 6 months follow-up.

^d^ICD: International Classification of Diseases.

^e^2 years before treatment start to treatment start.

^f^The systematic psychological treatments included the following national treatment provision codes: DU008, DU009, DU010, DU011, DU013, DU020, DU043, DU113, DU118, DU119, and DU120.

### Outcomes

#### Severity of Gambling Symptoms

GD symptoms were measured using the Gambling Symptom Assessment Scale (GSAS) [34], which served as the primary outcome according to the study protocol [32]. The GSAS is a 12-item self-report measure to assess the severity of gambling symptoms over the past week, aiding clinicians and researchers in tracking symptom progression during treatment. The GSAS has been found to be sensitive to treatment-related change [34]. The GSAS was administered with each iCBT module during treatment. No equivalent measure was available from the TAU arm of the cohort sample.

#### Gambling Activity

Gambling during the iCBT treatment was measured during each module using the Timeline Followback method [35]. Due to the low prevalence of gambling observed during treatment, along with high prevalence of reported use of stimuli control techniques, we opted to analyze gambling habits as a binary variable (any or none). No equivalent measure was available from the TAU arm of the cohort sample.

#### Negative Effects and Treatment Satisfaction

Any negative effects of the iCBT program were measured in conjunction with the last module, using the Negative Effects Questionnaire [36]. Relative occurrence of each reported negative effects is reported, along with distribution of attribution (treatment itself or other causes). Treatment satisfaction was measured at the same time using the Client Satisfaction Questionnaire (CSQ)–8 [37]. Furthermore, a tailored evaluation form was included with which patients rated which components of the treatment program they found helpful. No equivalent measures were available from the TAU arm of the cohort sample.

#### Registry Outcomes

From raw, timestamped data on care events, care commitments, and medication prescriptions, a set of registry-based outcomes were created using a stepwise procedure. First, an index date for treatment onset was created using the timestamp of relevant treatment provision codes. To avoid bias stemming from presumed differences in treatment duration between iCBT and TAU arms, patients in both arms were assigned a second index date corresponding to 6 months after treatment onset. Next, registry outcomes suggesting continued need for psychiatric treatment were computed using timestamped event data: new treatment onset within addiction care in Stockholm (GD-specific or not), new prescription of psychiatric medication, new enrollment in psychiatric inpatient care (GD-specific or not), and a registry entry indicative of social service involvement (in Sweden, health care and social services have a shared responsibility for providing treatment for GD). Examining both GD-specific and other treatment occurrences served to both control for detection bias (eg, systematic differences in tagging care event with the relevant diagnosis code), and to widen the scope of the outcome to cover, for example, psychiatric comorbidity (detailed outcome definitions in Table S2 in Multimedia Appendix 1 [32,33]).

### Statistical Analyses

To examine within-group change in the iCBT cohort during participants’ (varying) treatment windows, we modeled GSAS scores and gambling activity as a function of assessment occasion, the latter captured by a numeric variable (0-10) corresponding to the module. Since program composition changed during the cohort data collection period (10 modules being collapsed into 9), normalization of the time variable was required across program versions. While we initially considered making full use of the linear time variable by including noninteger timepoints, we opted instead to include all available data with the original sequence-based time integers but also to cap the primary end point at module 9. This decision was in part guided by the finding that only 1 patient undergoing the 10-module version reported 10 measurements. Changes during the treatment window were modeled using mixed-effects models (lmer and lmerTest) [38], either linear (GSAS) or logistic (any gambling), including random intercepts and slopes. GSAS within-group effect sizes were calculated using the standard Cohen *d* equation by extrapolating means from the mixed models (ensuring compliance with intention-to-treat and equal n) and observed SDs at the corresponding end point.

Registry outcomes were (retrospectively) gathered continuously (ie, not at fixed timepoints), and were therefore analyzed using survival analysis [39]. To adjust for the increased presence of psychiatric severity in the age- and sex-matched comparison patients (Table 1), survival analyses were run using both the complete TAU sample, as well as a matched subsample. This subsample was created using a propensity score matching procedure, using the 1:1 nearest neighbor method (MatchIt R package) [40], with past diagnoses and care as separate predictors.

### Ethical Considerations

All stages of the study were approved by the Swedish Ethical Review Authority (#2019-05479 and #2022-04987-02). All feasibility participants provided informed consent to data sharing for research purposes.

## Results

### Treatment Engagement

In the total iCBT cohort, patients sent an average of 5.87 (SD 6.10, median 4, range 0-28) messages to their therapists and began on average 4.16 (SD 3.10) modules, defined as completing at least 1 exercise per module (Figure S1 in Multimedia Appendix 1 for histogram [32,33]). The mean number of iCBT weeks during the treatment window was 11 (SD 7, range 0-26) weeks. A total of 6 therapists provided the iCBT treatment. The therapists were not randomized to patients, but assigned patients based on availability, that is, quasi-random. For the total TAU cohort, the mean treatment duration during the treatment window was 15 (SD 7, range 0-26) weeks. On average, patients received 6.34 (SD 4.5) treatment sessions face-to-face.

### Change in Symptoms and Gambling Activity

**Figure 1:**
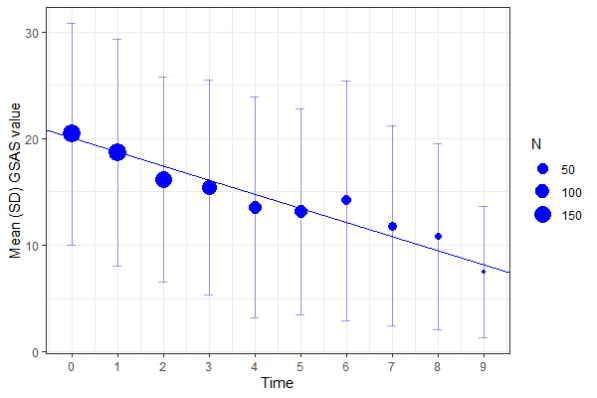
Change in gambling disorder (F630) symptoms during the internet-based cognitive behavioral therapy (n=188). GSAS: Gambling Symptom Assessment Scale.

Outcome measures were available for 188 (out of 218) patients who began the iCBT program. A statistically significant reduction in GSAS scores during the treatment window was observed (B=–1.33, SE=0.17, *P*<.001), corresponding to a large within-group Cohen *d* effect size, *d*=1.39. Visualization of change in symptoms are shown in Figure 1. No change in gambling activity was observed (*P*=.68). However, the average occurrence of any gambling during the treatment window was low, reported on only 19.4% of assessments, consistent with high rates (86.4%) of used stimuli control techniques (eg, the Spelpaus.se self-exclusion registry) during treatment.

A post hoc, exploratory analysis examined whether having any care event-marked comorbid psychiatric diagnosis moderated the change in GSAS score during treatment. A significant time×subgroup interaction effect (B=1.33, SE=0.67, *P*=.048) was found, revealing that not only did the comorbidity subgroup have a different slope, but also no significant change over time at all, despite similar intercepts (B=–0.42, SE=2.67, *P*=.88). Subsequent analyses revealed that the comorbidity subgroup also engaged in significantly less iCBT modules (ΔM=–1.63, *P*=.01)

### Negative Effects and Treatment Satisfaction

The assessment of negative effects was completed by 30 patients at the end of the iCBT program, and 20 patients reported at least 1 negative effect, with an average of 3.05 (SD 2.28) negative effects reported per patient. The most commonly reported negative effects included increased adverse emotional experiences such as unpleasant memories, stress, anxiety, or negative emotions. Patients attributed an average of 72% of the reported negative effects to the iCBT program and 28% to other circumstances, but this differed substantially between specific negative effects (100%-0% attribution). In addition, 2 patients reported suicidal thoughts, both unrelated to the iCBT (Table 2). Regarding treatment satisfaction, patients rated the iCBT program with a posttreatment mean rating of 27.9 (SD 2.94; n=32) out of 32 on the CSQ-8. In a separate evaluation form, patients (n=25) reported which specific useful treatment components they found useful, the most prevalent being reading about gambling-related loss of control and common reactions, reading about why people get stuck in gambling problems, and doing behavioral exercises when thinking about chasing losses (Table S3 in Multimedia Appendix 1 [32,33]).

**Table 2:** Self-reported negative effects of treatment in cohort sample.

Type of negative effect (NEQ^a^)	Reported by proportion of patients (total n=20), %	Attributed to	
		iCBT^b^, %	Other circumstances, %
Unpleasant memories resurfaced	80	94	6
I did not always understand my treatment	40	88	12
I experienced more anxiety	35	57	43
I experienced more hopelessness	35	86	14
I became afraid that other people would find out about my treatment	25	80	20
I felt like I was under more stress	20	50	50
I felt more worried	20	50	50
I started feeling ashamed in front of other people because I was having treatment	15	67	33
I got thoughts that it would be better if I did not exist anymore and that I should take my own life	10	0	100
I had more problems with my sleep	5	0	100
I experienced more hopelessness	5	0	100
I stopped thinking that things could get better	5	0	100
I did not always understand my therapist	5	100	0
I felt that the treatment was not motivating	5	0	100

^a^NEQ: Negative Effects Questionnaire [36].

^b^iCBT: internet-delivered cognitive behavioral therapy.

### Registry Outcomes

**Figure 2:**
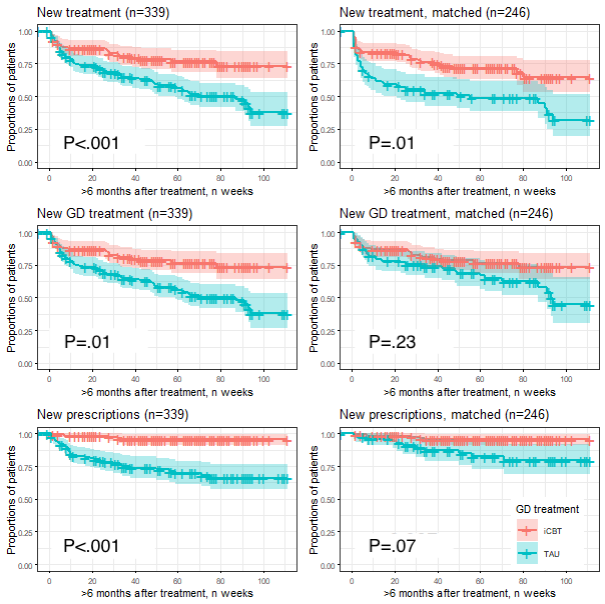
Survival analyses: new treatments and prescriptions. GD: gambling disorder (F630); iCBT: internet-delivered cognitive behavioral therapy at the Stockholm Addiction eClinic; TAU: treatment-as-usual, that is, systematic psychological treatment delivered face-to-face at any of the outpatient clinics at the Stockholm Center for Dependency Disorders.

Survival analyses revealed that patients in the iCBT arm, compared with the full TAU arm, were significantly less likely to re-engage in outpatient addiction care, GD-specific (*P*=.01) or not (*P*<.001), less likely to receive new psychiatric prescriptions (*P*<.001), less likely to be enrolled in psychiatric inpatient care (*P*=.01), and to show care events indicative of contact with social services (*P*=.04). When the iCBT arm was compared with the matched TAU subsample, none of the differences in outcomes remained significant, except non–GD-specific re-engagement in outpatient addiction care (*P*=.01) (Figure 2 and Table 3). The figure shows survival analyses of re-engagement in outpatient treatments and prescriptions of psychiatric medications in routine addiction care in 2 GD treatment samples; a total sample (n=339) that received either iCBT (n=123) or TAU (n=216) and a subsample (n=246) matched on comorbidity that received either iCBT (n=123) or TAU (n=213).

**Table 3:** Registry outcomes 6 months after treatment start.

Outcomes			iCBT^a^ (n=123), n	TAU^b^ (n=216), n	Matched TAU (n=123), n	Chi-square^c^ (*df*)	*P* value	
**Psychiatric inpatient enrollment**								
	**Any diagnosis**		0	14	5	—^d^	—	
		iCBT vs TAU	—	—	—	6.76 (2)	.01	
		iCBT vs matched TAU^e^	—	—	—	3.27 (2)	.07	
	**Gambling Disorder**		0	0	0	—	—	
		iCBT vs TAU	—	—	—	—	—	
		iCBT vs matched TAU	—	—	—	—	—	
**Involvement of social services**			1	13	2	—	—	
	iCBT vs TAU		—	—	—	4.13 (2)	.04	
	iCBT vs matched TAU		—	—	—	0 (2)	≥.99	

^a^iCBT: internet-delivered cognitive behavioral treatment at the Stockholm Addiction eClinic.

^b^TAU = treatment-as-usual, that is, systematic psychological treatment delivered face-to-face at any of the outpatient clinics at the Stockholm Center for Dependency Disorders.

^c^Pearson chi-square test with Yates continuity correction.

^d^Not available.

^e^Matched TAU: treatment-as-usual, sample matched on comorbidity.

## Discussion

### Principal Findings

This paper describes the introduction of a newly developed iCBT program for GD provided within routine addiction care.

Patients who began the program were found to significantly decrease GD symptoms during the treatment window, with large within-group effect sizes in both the feasibility and registry samples. These effects were roughly equivalent to those reported in trials including participants with problem gambling from the general population [15] and iCBT with weekly telephone support [41]. Patient engagement in the iCBT modules was roughly comparable to other iCBT gambling trials in the general population, for example, studies by Dowling et al [42] and Magnusson et al [43]. The iCBT program was rated high in terms of treatment satisfaction; however, it should be noted that this score may not be fully representative. Regarding registry-based outcomes, iCBT patients showed overall similar outcomes once psychiatric severity was controlled for. Since our cohort sample lacked randomized allocation to the 2 arms, the findings of this study should not be interpreted to suggest causality. Although the final TAU sample only included patients who had undergone a systematic form of psychotherapy, it should be emphasized that the psychotherapeutic content differed between conditions, and that some face-to-face TAU treatments (eg, psychodynamic therapy) are not recommended for GD in national guidelines [12]. These study design limitations notwithstanding, the totality of our findings does show that the iCBT program did not merely serve to funnel patients into regular care, but appears to be effective in reducing GD symptoms in its own right. Of importance, while measures of gambling activity and GD symptoms were only collected during treatment (entailing missing data among those who never began treatment), the registry outcomes included all Stockholm-residing patients that enrolled in the iCBT, meaning that the included survival analyses were in full compliance with the intention-to-treat principle and thus provide an unbiased estimate of effectiveness. Ratings of treatment satisfaction were congruently high, although this measurement bears the same limitation of representativity as above. Furthermore, using a tailored instrument like the Internet Evaluation and Utility Questionnaire [44] would likely have been a more suitable choice than the general CSQ, as it includes evaluation items specific to internet-delivered treatment format.

Some patients reported negative treatment effects such as unpleasant memories, stress, anxiety, or negative emotions. This was an expected result, since the iCBT program actively encouraged patients with GD to confront gambling-related situations and manage them differently; similar negative effects have been reported for CBT in the context of anxiety [36]. It should be noted that negative effects were only reported by a small group of patients who completed large parts of the program, and the overall low occurrence of negative effects may thus not generalize to the entire treatment population. Nonetheless, no patient reported that the iCBT failed to produce positive results, or that gambling-related deterioration occurred. Furthermore, no GD patient reported any negative effects related to the iCBT exercises. This finding is noteworthy, as the behavioral exercises emphasize increased control in gambling-related situations, in some cases even involving gambling for small amounts of money under controlled circumstances (including asynchronous therapist support). In addition, 2 patients reported suicidal thoughts, although these were not attributed to the iCBT program itself. A high prevalence of suicidal thoughts and behaviors among patients with GD has been reported in several previous studies, for example, studies by Black et al [3] and Håkansson and Karlsson [45]. This study contributes to this knowledge base, suggesting the importance of continuous clinical monitoring and assessment of suicidal thoughts and behaviors among treatment-seeking patients with GD. To reduce future attrition of the reporting of negative effects, assessments of negative effects could be administered earlier in treatment.

From a clinical perspective, the field of GD research is still partly in its infancy, and a prioritized challenge related to treatment involves the inclusion of disorder-specific interventions grounded in basic behavioral research. In this study, we have reported the introduction and evaluation of a newly developed iCBT program, which emphasizes 4 key psychological processes relevant to GD, that are access to money, reward expectation, chasing behaviors, and the gambling zone. The iCBT behavioral exercises targeting these GD processes appear to have been well-received, as indicated by a survey distributed toward the end of the treatment. However, it is important to note that this finding may not generalize to the entire sample due to the high attrition rate, and it is reasonable to hypothesize that patients who appreciated these exercises were more likely to complete the program in its entirety. As with mapping of negative effects, future iterations of the program should aim to include this survey in an earlier module to reduce missing data. Exploring how early experiences with the program influence subsequent treatment, engagement should be considered an important topic for future research, as well as the development of high-accuracy prediction models to identify who is likely to benefit from treatment. Although beyond the scope of this study, it is worth emphasizing that the iCBT program was designed to build on a simple, delimited set of theoretically derived interventions. This design enables future research on specific mediators and treatment mechanisms, a recommended feature for clinical GD trials [46].

A final GD treatment-related challenge concerns psychiatric comorbidity. Throughout the clinical procedure used in this study, caution was taken not to exclude patients based on comorbidity. However, we noted that the prevalence of psychiatric comorbidity seemed lower in the iCBT sample, compared with the TAU sample, as well as in comparison to prevalence estimates among treatment-seeking patients with GD reported elsewhere (Dowling et al [26] shows a meta-analysis). This is likely due to a study design detail, with comorbidity diagnoses being extracted from linked care events (to ensure up-to-date and still applicable diagnoses) at an Addiction eClinic that does not typically offer treatment for comorbidity. However, since moderating effects were indeed observed, even with a smaller subgroup sample, there is no reason to believe that this post hoc analysis was underpowered. Findings from this study indicate that patients with GD with psychiatric comorbidities can benefit from additional targeted treatment interventions, although it should also be noted that face-to-face treatments for GD in general have been found effective, even when additional disorders were present [47]. Whether the potential impact of comorbidity is inherently linked to the internet format, which places greater demands on the patients’ abilities to independently plan and engage with the treatment material, remains unknown. It should also be stressed that the subgroup with comorbidity that was identified, likely represents those with more severe comorbidity; to what degree moderation can be observed also at lower severity levels, also remains unknown. In sum, given the high rates of comorbidity among patients with GD, elucidating the moderating and mediating effects of this on GD outcomes should be considered a research priority.

The iCBT program was introduced and made nationally available to treatment-seeking patients, through the TAS platform which is integrated into the national health care guide at 1177.se. Treatment dissemination is a crucial challenge, and we conclude that the TAS platform is a viable technical infrastructure to distribute internet-delivered psychological treatments among patients in the health care system. Importantly, the fact that measurements were collected from the same platform used for treatment, entails that there is little reason for patients who drop out of treatment to continue reporting outcomes, making it difficult to conduct true intention-to-treat analyses using this data source. Another issue that became obvious, unrelated to the TAS platform but with obvious implications for design of future studies, was the low rates of any gambling activity, and the concurrent high rates of used stimuli control techniques. Given that the national gambling self-exclusion registry Spelpaus.se, at time of writing, has over 100,000 individuals signed up and is explicitly referred to as a “First Aid” for GD, this is not unexpected. It also illustrates the necessity of informed study design considerations when attempting to estimate effectiveness of GD treatments.

### Strengths and Limitations

Strengths of this study include a comprehensive cohort design with side-by-side reporting of findings from 2 different samples, the use of a matched TAU comparison sample, and registry-based outcomes covering a follow-up period of up to 2 years, with no (theoretical) missing data during this period. Since the study was conducted within the very setting and under the same conditions that continue to apply, we are convinced that our findings have high generalizability. The study also has several limitations, both in terms of design and individual measures. Regarding outcomes collected during the iCBT treatment window, these were only available (including missing data estimation) from those who actually began treatment and completed at least 1 measure (188/218); generalizability is thus limited to patients who initiated treatment, not all patients allocated to treatment. Regarding registry outcomes, the ethical approval did not cover registry linkage with the Swedish address registry, entailing that changes in region of residence during the follow-up period could not be taken into account, constituting a possible source of bias under the assumption that the 2 arms are differentially likely to change region of residence. Only having Stockholm-residing iCBT patients be matched to TAU patients should mitigate this issue, at least in part. However, patients in Sweden are free to seek treatment in other regions, including the iCBT program evaluated here. It should also be noted that while registry data does not bear missing data in the typical sense, since all health care contact by law needs to be recorded, the registry data examined in this study does not provide a complete view since it does not cover visits to nonregional health care interventions, such as contact with social services or gambling support lines, or participation in peer-support groups. Another important limitation is that the registry follow-up data did not include measures of actual gambling, only treatment-seeking (including for gambling). Such data could have been collected by, for instance, sending out invitations to patients to respond to a survey; alas, resources for this were not available. In theory, it would also be possible to extend the registry follow-up to also include enrollment in the Spelpaus.se self-exclusion register; such linkage should be attempted in future research. A final limitation is that propensity score matching could only be performed on registry variables, which did not include potentially influential factors such as education. Furthermore, the treatment provided to the TAU cohort was not necessarily matched to the iCBT cohort in terms of treatment structure or content. As in any nonrandomized observational study, causal interpretations of difference should hence be done with great caution, yet we argue that findings of outcomes in the TAU cohort nonetheless constitute a meaningful clinical benchmark with which to compare newer treatments.

### Conclusions

A newly developed iCBT program for GD, introduced into routine addiction care, was feasible, well-received by patients, and was associated with the expected decrease in symptoms during the treatment window. The study highlights the need for GD treatment development in tandem with ongoing research and monitoring of treatment effects, especially in the context of psychiatric comorbidity among patients with GD. The findings also underscore the importance of careful study design and technical considerations when introducing internet-based treatments into the routine care setting. Future randomized controlled trials and studies of iCBT treatment mechanisms, and the moderating effects of psychiatry comorbidity, are warranted.

## Appendix

Multimedia Appendix 1 Feasibility Study.

## Abbreviations

CBT: cognitive behavioral therapy
CSQ: Client Satisfaction Questionnaire
DSM-5: Diagnostic and Statistical Manual of Mental Disorders, fifth edition
DSM-IV: Diagnostic and Statistical Manual of Mental Disorders, fourth edition
GD: gambling disorder
GSAS: Gambling Symptom Assessment Scale
iCBT: internet-delivered cognitive behavioral therapy
TAS: Treatment and Support
TAU: treatment-as-usual

## Data Availability

The data sets generated and analyzed during this study are not publicly available due to patient confidentiality and cannot be shared without permission from the health care provider, but are available from the corresponding author on reasonable request.
