# Supplementary material for: Introducing and Evaluating the Effectiveness of Online Cognitive Behavior Therapy for Gambling Disorder in Routine Addiction Care: Comparative Cohort Study
Source: J Med Internet Res. 2024 Sep 18;26:e54754. doi: 10.2196/54754 (PMC11447416; doi:10.2196/54754)
Supplement: Multimedia Appendix 1 [file jmir_v26i1e54754_app1.docx]

**Introduction**

The iCBT program was made available using the Swedish national eHealth platform (*Stöd och behandling*, Treatment and Support [TAS] platform). The TAS platform is integrated into the national healthcare guide *1177.se*, allowing prospective patients to conveniently access both information, patient services and online treatments within the same platform. Access required a bank-issued digital ID, after which notifications by text message and email could be enabled by each individual user. As part of the introductory process, four eClinic psychologists participated in a half- day training workshop covering GD as well as the content of the iCBT program; they also participated in clinical supervision sessions during the treatment introduction, all held by author OM.

**Recruitment and procedure**

Pre-trial registration for an introductory feasibility study was completed on May 10, 2019, at Clinical trials.gov (NCT03946098), and a study protocol was published [1]. Study recruitment began in October 2019, and ended in July 2020 at a pre-set date, at which point the intended sample size of n = 25 had not been met. The last follow-up measure was administered in February 2021. Initially, recruitment of patients occurred exclusively through formal clinical referrals to the Addiction eClinic from one of eight outpatient clinics at the Stockholm Center for Dependency Disorders. Information about the availability of iCBT was also provided in a written pamphlet, which included the Problem Gambling Severity Index (PGSI) [33] screener to facilitate referrals. Patients showing interest in iCBT were referred clinically to the Addiction eClinic, where they met with a physician for assessment of eligibility, after which a decision was made regarding whether iCBT was suitable. The inclusion criteria were: (a) being at least 18 years old, (b) living in Sweden, (c) having the ability to work with online treatment material on one’s own, and (d) being able to read and write Swedish. Patients were excluded from the study if they (a) fulfilled criteria for ongoing manic episode, since this is an exclusion criterion for GD 7; or (b) were undergoing parallel CBT treatment for problem gambling. Since participants were recruited from a clinical setting, it was not possible to accurately log how many were exposed to the offer of iCBT but turned it down. In January 2020, five months after recruitment to the feasibility study started, an additional recruitment path opened when the iCBT gambling program was made nationally available via the problem gambling information page on the website of the national healthcare guide (*1177.se*). Through a link, treatment-seeking gamblers accessed information about the treatment at the Addiction eClinic website and were instructed to register their interest by completing a form on the TAS platform. Prospective patients were then contacted by the Addiction eClinic for clinical assessment of eligibility, which was conducted by a physician either on-site at the Addiction eClinic in Stockholm, or via secure videocall through a smartphone application provided by the Region Stockholm healthcare. Eligible patients were given access to the iCBT program through the TAS platform, where they were asked whether they were willing to provide informed consent to participate in this feasibility study. See Supplementary Table 1 for participant characteristics.

Table S1. Participant characteristics, feasibility study.

|  | iCBT feasibility  sample  (n= 23) |
| --- | --- |
| Age, M (Sd) | 32.8 (11.95) |
| Gender, n (%) |  |
| Men | 16 (70%) |
| Women | 7 (30%) |
| Diagnostic codes^b^ |  |
| Gambling disorder (F630) | 19 (83%) |
| Other ICD diagnoses | 3 (13%) |

Note:

iCBT = Internet-delivered cognitive behavioral treatment at the Stockholm Addiction eClinic.

**Change in symptoms and gambling activity**

Outcome measures were available for n=20 (of n=23) patients who began the iCBT program. A statistically significant reduction in GSAS scores during the treatment window was observed (B = -1.39, SE = 0.30, P = .0165), corresponding to a large within-group Cohen’s d effect size of d = 1.69. See Supplementary figure X below for visualization of change in symptoms. A significantly decreasing odds ratio of any gambling was observed (P= .003, random slopes dropped due to convergence issues). However, the average occurrence of any gambling during the treatment window was low, 9.1% of assessments, consistent with high rates (86.4%) of employed stimuli control techniques (e.g., the Spelpaus.se self-exclusion registry) during treatment.

Figure S1. Change in GD symptoms during the iCBT (n=20).


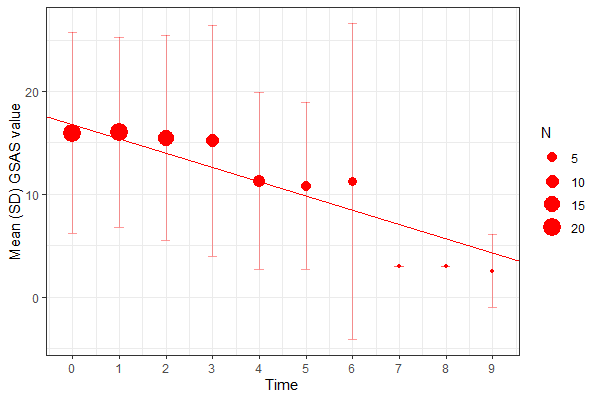


GD = Gambling Disorder (F630)

**Treatment evaluation and modifications**

After the feasibility study was completed, a structured process was initiated to evaluate the content and structure of the iCBT program from a treatment provider perspective. Experiences from psychologists at the Addiction eClinic were gathered in internal meetings and at a workshop held by OM, after which revisions were made to the iCBT program, and published on the treatment platform (see below) on November 1, 2021. The treatment modifications included collapsing the iCBT program into 9 modules, adding information and exercises related to the self-exclusion registry Spelpaus.se and to gambling on the stock market, adding additional safety information and checkpoints to ensure that patients did not engage in exercises that were too difficult for them early in the program, minor revisions to homework assignments, and revising the maintenance plan and evaluation of treatment goals.

**Conclusions**

Clinical quality assurance work and preliminary effects indicated promising results during the pilot phase. Based on these findings, a decision was made to continue provision of the iCBT within the framework of regular specialist addiction care.

Table S2. Registry variable definitions.

| Variables | Definition |
| --- | --- |
| *Treatments* |  |
| iCBT | Internet-delivered cognitive behavioral treatment at the Stockholm Addiction eClinic, with the following national treatment provision code: DU* |
| TAU | Systematic psychological treatments including the following treatments and national treatment provision codes: Psychodynamic therapy (DU008), other specified psychological treatment (DU009), cognitive therapy (DU010), cognitive behavioral therapy (DU011), mentalization-based treatment (DU013), systematic psychological treatment (DU020), other psychodynamic therapy (DU043), systematic supportive conversation (DU113), motivational interviewing (DU118), relapse prevention (DU119), motivational enhancement therapy (DU120). |
| *Diagnostics* |  |
| Gambling Disorder | A diagnosis of Gambling Disorder 2 years before treatment start to 6 months follow-up, with the following national treatment provision code: F630. |
| Other ICD  categories |  |
| Mental and  behavioral  disorders due  to use of  psychoactive  substances | Psychiatric diagnoses 2 years before treatment start to 6 months follow-up, including the following national treatment provision codes: F10 to F19 |
| Mood/affective  disorders | Psychiatric diagnoses 2 years before treatment start to 6 months follow-up, including the following national treatment provision codes: F30 to F39. |
| Neurotic, stress-  related and  somatoform  disorders | Psychiatric diagnoses 2 years before treatment start to 6 months follow-up, including the following national treatment provision codes: F40 to F49. |
| Disorders of  personality  and behavior in  adult persons | Psychiatric diagnoses 2 years before treatment start to 6 months follow-up, including the following national treatment provision codes: F60 to F69. |
| *Outcomes* |  |
| New treatment  onset within  addiction care  in Stockholm |  |
| Due to any  diagnosis | Outpatient (any) care events six months after treatment start index. |
| Due to  Gambling  Disorder | Outpatient care events six months after treatment start index, including the following national treatment provision code: F630. |
| New psychiatric  inpatient  enrollment |  |
| Due to any  diagnosis | Psychiatric inpatient enrollment six months after treatment start index. |
| Due to  Gambling  Disorder | Psychiatric inpatient enrollment six months after treatment start index, including the following national treatment provision code: F630. |
| New prescription  of psychiatric  medication | Prescription of (any) psychiatric medication in addiction care six months after treatment start index. |
| New involvement  of social services | Network meetings with staff from social services six months after treatment start index, including the following national treatment provision codes: AU124, AU125, ZV509. |

iCBT = Internet-delivered cognitive behavioral treatment at the Stockholm Addiction eClinic.

ICD = International Statistical Classification of Diseases and Related Health Problems

TAU = Treatment as usual, i.e., systematic psychological treatment delivered face-to face at any of the outpatient clinics at the Stockholm Center for Dependency Disorders.

Table S3. Helpful iCBT components.

| Parts of the  iCBT program | Reported as helpful by patients  (total n=25) |
| --- | --- |
| Reading about gambling-related loss of control and common  reactions | n=25 (100%) |
| Reading about why people get stuck in gambling | n=24 (96%) |
| Doing behavioral exercises when I think about chasing losses | n=21 (84%) |
| Reading about how other individuals have worked with different  parts of the treatment | n=20 (80%) |
| Doing behavioral exercises when I get caught in the pitfalls of  gambling (the gambling zone) | n=18 (72%) |
| Doing behavioral exercises when I have the opportunity to gamble  and experience expectancy | n=16 (64%) |
| Setting goals for the treatment | n=16 (64%) |
| Seeking out and writing down my gambling situations | n=16 (64%) |
| Doing other types of behavioral exercises | n=10 (40%) |

iCBT = Internet-delivered cognitive behavioral treatment at the Stockholm Addiction eClinic.

Figure S2. Distribution of number of begun modules in iCBT cohort sample.


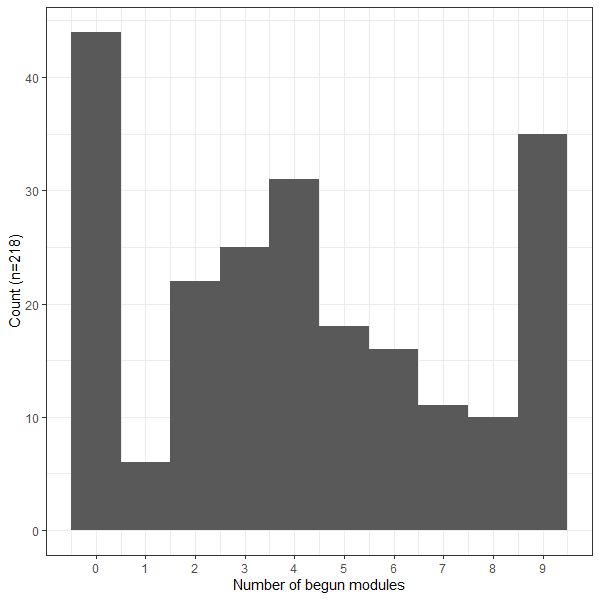


iCBT = Internet-delivered cognitive behavioral treatment at the Stockholm Addiction eClinic.
